# Cortisol, anxiety and cognitive responses to trier social stress test: The first multiple levels assessment of the rdoc “system for social process” in eating disorders

**DOI:** 10.1192/j.eurpsy.2021.327

**Published:** 2021-08-13

**Authors:** V. Ruzzi, G. Cascino, M. Raia, V. Sollo, A.M. Monteleone

**Affiliations:** 1 Department Of Psychiatry, University of Campania “Luigi Vanvitelli”, Naples, Italy; 2 Department Of Medicine Surgery And Dentistry - Section Of Neuroscience, University of Salerno, Salerno, Italy

**Keywords:** psychosocial stressor, cortisol reactivity, emotional distress, eating disorders

## Abstract

**Introduction:**

Social dysfunction is a putative risk and maintaining factor for Eating Disorders (EDs).

**Objectives:**

We aimed to assess biological, emotional, and cognitive responses to a psychosocial stressor, in order to provide a multilevel investigation of the RDoC social process system in EDs.

**Methods:**

Cortisol response to Trier Social Stress Test (TSST) was measured in 105 subjects: 35 women with anorexia nervosa (AN), 32 with bulimia nervosa (BN) and 38 healthy women. In a subgroup of them (23 AN, 21 BN, and 25 control women) anxiety, hunger, and desire to eat throughout the TSST were also rated.

**Results:**

Compared to healthy women, AN and BN women showed reduced cortisol reactivity that disappeared after controlling for trait anxiety and ineffectiveness. They also displayed increased anxiety response, while only people with AN reported greater decrease in hunger and desire to eat. Baseline ineffectiveness predicted post-stress body dissatisfaction through the mediation of post-stress anxiety while no significant correlations were found between cortisol and anxiety, hunger, or desire to eat responses

**Conclusions:**

People with EDs are characterized by blunted cortisol reactivity and greater anxiety, hunger, and desire to eat responses to a psychosocial stressor. We show a relationship between socio-emotional distress and ED-related attitudes without an association between biological and emotional or cognitive changes. This study provides the first empirical and multilevel support to a deranged functioning of the RDoC “system for social process” in EDs.

**Disclosure:**

No significant relationships.

